# Preparation of Mg(OH)_2_/Calcined Fly Ash Nanocomposite for Removal of Heavy Metals from Aqueous Acidic Solutions

**DOI:** 10.3390/ma13204621

**Published:** 2020-10-16

**Authors:** Caili Wang, Jing Wang, Shaobin Wang, Runquan Yang, Huaifa Wang

**Affiliations:** 1 College of Mining Engineering, Taiyuan University of Technology, Taiyuan 030024, China; wangjing@tyut.edu.cn (J.W.); yangrunquan@tyut.edu.cn (R.Y.); wanghuaifa@tyut.edu.cn (H.W.); 2 State Environmental Protection Key Laboratory of Mineral Metallurgical Resources Utilization and Pollution Control, Wuhan University of Science and Technology, Wuhan 430081, China; 3 School of Chemical Engineering, The University of Adelaide, Adelaide 5005, Australia; shaobin.wang@adelaide.edu.au

**Keywords:** Mg(OH)_2_/calcined fly ash composite, heavy metals, acidic aqueous solution, wastewater, composite materials, powder technology

## Abstract

A magnesium hydroxide (MH)-modified calcined fly ash (CFA) nanocomposite (CFAMH) with core-shell structure was obtained with a heterogeneous nucleation method, and its application for removal of copper, zinc and nickel ions from aqueous acidic solution was studied. The microstructure and surface properties of CFA, CFAMH and MH powders were characterized by scanning electron microscopy (SEM), Brunauer-Emmett-Teller specific surface area (BET), X-ray diffraction (XRD) and Fourier translation infrared spectroscopy (FTIR), respectively. The preparation mechanism of CFAMH was discussed based on zeta potential and FTIR data. The results showed that nano-flake MH with thickness 13.4 nm was well coated on the surface of CFA. The specific surface area was increased from 2.5 to 31.0 m^2^/g. Si-O-Mg-OH bonds formed from the condensation of Si-OH and Mg-OH. The removal efficiency of heavy metals on CFAMH nanocomposite is higher than that of CFA and MH and follows an order of Cu^2+^ > Zn^2+^ > Ni^2+^. Solubility product constant (Ksp) is an important constant for the removal order of heavy metals on FA, CFAMH and MH. CFAMH nanocomposite can be a cheap material for removing heavy metal ions from acidic wastewater.

## 1. Introduction

The discharge of acid and toxic heavy metals such as copper(Ⅱ), nickel(Ⅱ) and zinc(Ⅱ) into the environment is accelerating annually with the rapid development of economics and industries, leading to serious environmental pollution and health problems to the human body for their recalcitrance and persistence [1]. Therefore, removal of these heavy metals from contaminated water has become an important issue.

To date, different techniques, including adsorption [2], ion exchange [3], membrane processes [4], chemical precipitation [5], photocatalytic degradation [6], reverse osmosis [7], coagulation [8], solvent extraction [9], flotation [10] and advanced oxidation [11,12], have been applied for adsorbing heavy metal ions from aqueous media. Among these methods, adsorption was considered as one of the most effective techniques owing to its low-cost, environmentally friendly, simple operation and regeneration of the adsorbents by suitable desorption process [13]. Novel nanomaterials are constantly applied for removal of heavy metals for their superior performance. The most commonly used nanomaterials are metal-organic framework (MOFs) [14], nanoscale zero-valent irons (NZVI) [15], mxenes [16], magnesium oxide [17] or magnesium hydroxide [18] and other inorganic nanoparticles [19,20]. Among which, magnesium hydroxide (MH) is a promising environmentally friendly water treatment adsorbent for pH value no more than 9, easy operation, non-toxic, non-harmless and dissolving slowly in water. Besides, it has functional groups such as hydroxyl group as an active site for adsorption. However, an extra process for separation including long-time centrifugation or filtration is needed after the adsorption use due to its nano-size. The high cost of nano-Mg(OH)_2_ also limits its application. Therefore, how to accelerate the sedimentation and filtration rate of magnesium hydroxide is a key problem when it is used as an adsorbent of heavy metal ions in sewage. Depositing nano-size materials on the surface of micro-size minerals to prepare core-shell composite materials is considered as a useful process to solve this problem as the coating materials’ size will be increased.

Adsorption of heavy metals with low-cost adsorbents from contaminated water have been paid much attention [21]. For adsorption with a solid waste, fly ash (FA) is widely studied because of its ready availability and inexpensiveness [22]. However, the adsorptive ability of fly ash is limited. Increasing the specific surface area of fly ash can increase its removal efficiency for heavy metal ions [23,24].

Recently, many researchers have shown that coating inorganic nanomaterials on the surface of matrixes such as carbon or natural minerals could lead to increased adsorption abilities of the matrixes due to increased specific surface area and more active groups and solve the problem of nanomaterials for easy aggregation and difficult recycling [25,26]. Up to the best of our knowledge, MH-modified calcined fly ash (CFA) nanocomposite (CFAMH) has not been reported and used for the removal of heavy metal ions from acidic solution. Therefore, the present work aims to investigate the preparation of a CFAMH nanocomposite and its use for removal of heavy metals from aqueous solution.

## 2. Experimental Procedure

### 2.1. Materials

Fly ash (FA) was obtained from Shang Hai Ge Rui Ya Nano Materials Technology Co. Ltd. (Shanghai, China). FA was calcined in a muffle furnace at 815 °C in air for 2 h to obtain calcined fly ash (CFA). The chemical composition of FA is as follows: SiO_2_: 54.7%, Al_2_O_3_: 29.78%, Fe_2_O_3_: 4.06%, TiO_2_: 1.25%, CaO: 3.30% and loss on ignition (LOI): 3.26%. Magnesium sulfate (purity 98%), sodium hydroxide (purity 98%), copper nitrate (purity 99%), nickel nitrate (purity 99%) and zinc nitrate (purity 99%) were of analytical grade and purchased from Sigma-Aldrich (Shanghai, China) and used as received. Distilled water was used for preparation of solutions.

### 2.2. Preparation of CFAMH

**Figure 1:**
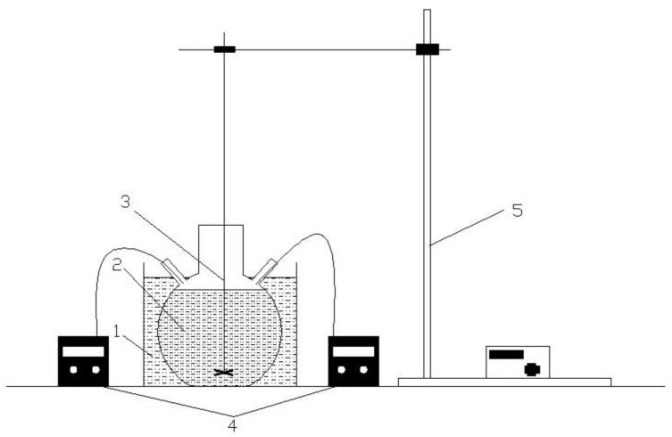
Schematic diagram of experimental device for preparing calcined fly ash coated with magnesium hydroxide (CFAMH) and magnesium hydroxide (MH). 1: Water bath, 2: CFA suspension, 3: Rotor, 4: Constant flow pump, 5: Iron support.

CFAMH nanocomposite was prepared as shown in Figure 1. CFA and water were put in the three-neck flask, stirred and heated; when the temperature reached 90 °C, the NaOH and MgSO_4_ solution were added simultaneously with the constant flow pump. The dosage of CFA to water is 1:5. The coating dosage of MH on the surface of CFA is 70%. The NaOH and MgSO_4_ solution concentration is 0.3 and 0.15 mol/L, respectively. The NaOH and MgSO_4_ solution were added simultaneously and the drops’ acceleration was 5 mL/min. The reaction temperature is 90 °C. The pH value was adjusted to 10 when NaOH and MgSO_4_ solution dropping was completed and reacted for a further 90 min. The suspension was filtered and repeated and washed with 1000 mL distilled water to remove the residual ions. The precipitate was then dried at 105 ± 3 °C for 24 h and ground to obtain a white powder. MH was prepared with the same method, without CFA.

### 2.3. Characterization

The surface morphologies of FA, CFA, CFAMH and MH were examined by JSM-35C scanning electron microscopy (Japan Electron Optics Laboratory Co., LTD, Akishima, Japan). The specific surface area of FA, CFA, CFAMH and MH were measured by JW-BK nitrogen sorption isotherm. X-ray diffraction (XRD) patterns of FA, CFA, CFAMH and MH were acquired with a Japan Rigaku MiniFlex600 X-ray diffractometer (Japan Rigaku Co., Ltd., Akishima, Japan) with a Cu X-ray tube (50 kV, 200 mA) over a 2θ range of 5–80° through a 0.02° rad soller slit and scanning speed of 6°/min at room temperature. Fourier-transform infrared spectroscopy was undertaken on a TENSOR27 spectrometer (German Bruker Co., Ltd., Karlsruhe, Germany). The zeta potential values of CFA and Mg(OH)_2_ under different pH values were detected with a JS94H zeta potential meter (Shanghai Zhongchen Digital Technology Co., Ltd., Shanghai, China).

### 2.4. Adsorption Studies

The adsorption experiments were performed as follows: a known weight of CFA, CFAMH and MH was added to conical flasks containing 25 mL of heavy metal solution with different initial concentrations (50, 35 and 20 mg/L) at different initial pH values (1, 2 and 4), at 25 °C. The conical flasks were shaken uniformly at 150 rpm for 10–1440 min. All the resultant solutions were centrifuged (7800 rpm, 10–20 min) to get the supernatant liquids for finally analyzing the concentration of copper, zinc and nickel ions by ICP-OES (PerkinElmer, Shanghai, China) on the optima 8300. The removal percentage of samples with each heavy metal ion was calculated.

## 3. Results and Discussion

### 3.1. SEM Observation

**Figure 2:**
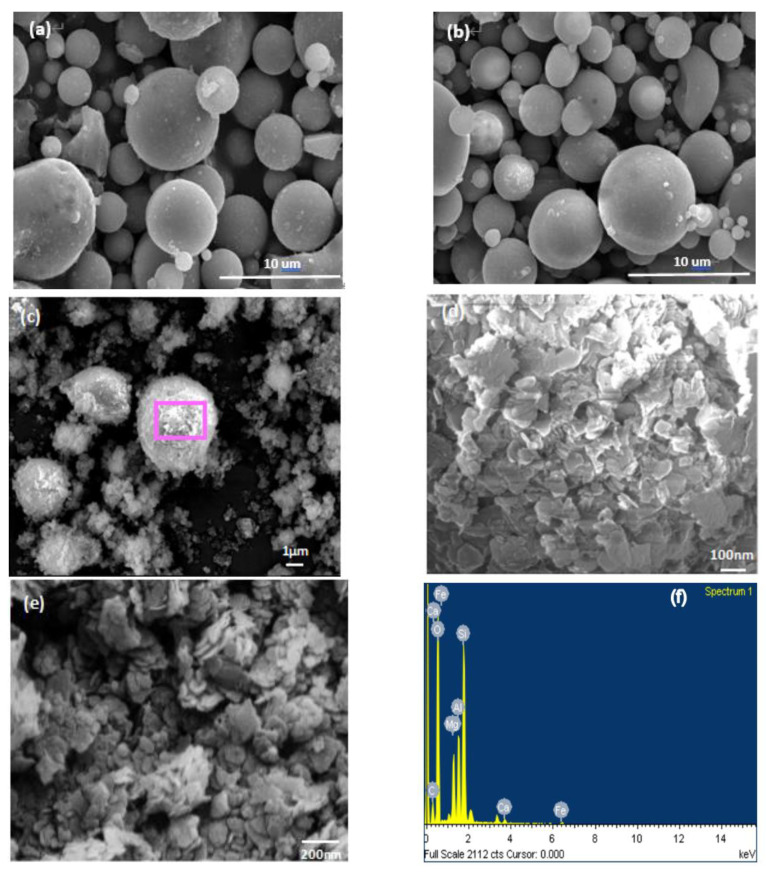
SEM photographs of (**a**) fly ash (FA), (**b**) calcined fly ash (CFA), (**c**) calcined fly ash coated with magnesium hydroxide (CFAMH) × 5000, (**d**) CFAMH × 50,000, (**e**) magnesium hydroxide (MH) × 25,000 and (**f**) EDS of CFAMH.

SEM micrographs of FA, CFA, CFAMH and MH, and energy dispersive spectrometer (EDS, Japan Electron Optics Laboratory Co., LTD, Akishima, Japan) CFAMH are shown in Figure 2. FA presents a spherical shape, and many unburned carbon particles are adhered to the surface (Figure 2a). CFA became smooth and still had good spherical shape after being calcined at 815 °C for 2 h (Figure 2b). The specific surface area of the CFA was decreased from 3.7 to 2.5 m^2^/g (Table 1), indicating that the unburned carbon was removed. CFAMH shows rough surface (Figure 2c). The average grain size of MH on CFA was about 8–15 nm (Figure 2d). Table 1 is the specific surface areas and pore characteristics of FA, CFA and CFAMH powders. It shows that the specific surface area was increased from 2.5 (CFA) to 31.0 m^2^/g (CFAMH) (Table 1). The MH aggregates show the nano-flaky morphology, and the average grain size of MH is obviously bigger than that coated on CFA (Figure 2e), suggesting that more active groups of MH were generated with the same method if coated on CFA. EDS shows that the weight of elements of CFAMH is C (14.69%), O (50.88%), Mg (5.96%), Al (7.76%), Si (18.63%), Ca (0.78%) and Fe (1.31%).

### 3.2. Particle Size Distribution

**Figure 3:**
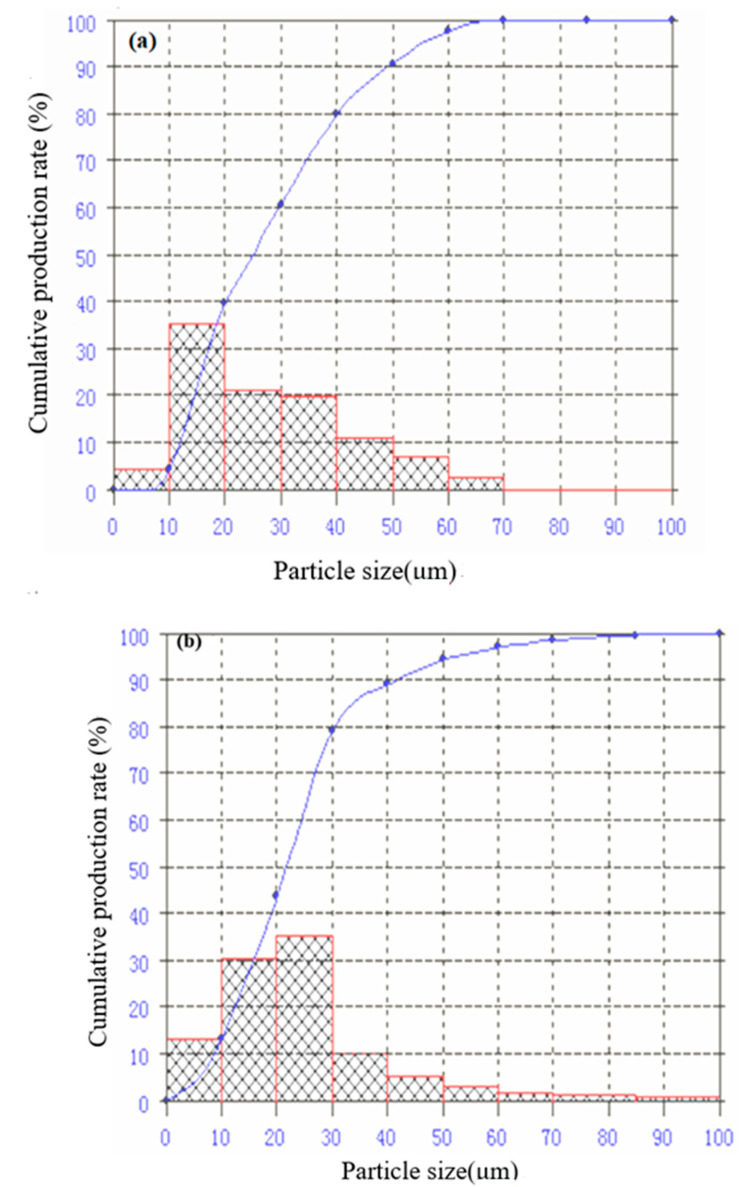
Particle size distribution of (**a**) CFA and (**b**) CFAMH.

Figure 3 is the particle size distribution of CFA and CFAMH. It can be seen that D_97_ increased from 58.74 to 62.08 μm, which is due to coating of MH.

### 3.3. XRD Analysis

**Figure 4:**
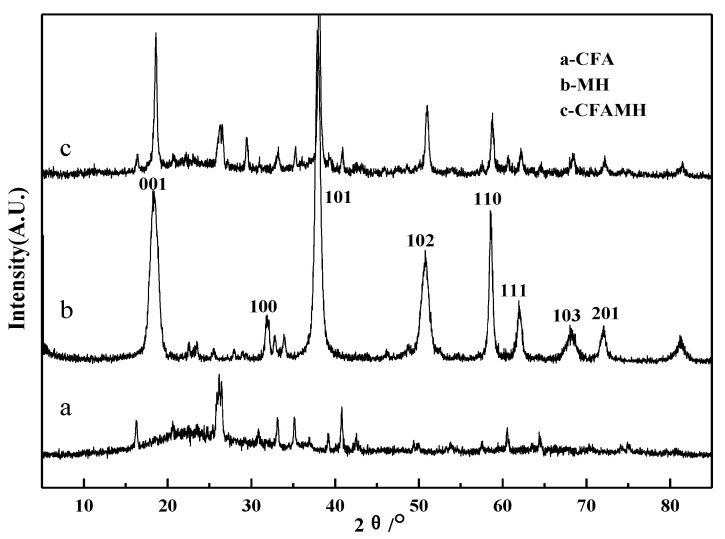
X-ray diffraction (XRD) spectrum of CFA, MH and CFAMH.

Figure 4 is the XRD spectrum of CFA, MH and CFAMH. The XRD spectrum of CFA is well with the one reported before [27]. The XRD of MH is referring to the Reference Code 441482 data. After coating with MH, CFA phase and MH phase are present in the composite. The patterns of CFAMH peaks are mostly similar to the CFA, and new peaks that appeared in the CFAMH belong to MH [28], indicating the CFAMH composite was successfully synthesized. The (001), (101), (102), (110), (111), (103) and (201) peaks of MH samples are obviously observed at 2θ = 18.60°, 38.10°, 51.02°, 58.80°, 62.18°, 68.36° and 72.18°, respectively. The peak of (101) face has the sharpest intensity, indicating that the crystal face in this direction is more complete. The average crystallite sizes of MH with different crystal face on CFAMH were calculated by the Scherrer equation (*D* = Kλ/(βcosθ)) and listed in Table 2 [27], where *D* is the average particle size of MH on CFAMH in different crystal faces, *k* = 0.94 is a coefficient, λ = 0.15418 nm is the X-ray wavelength, β is the full-width half-maximum (FWHM) of the CFAMH, θ is the diffracting angle, and h is the intensity at different crystal faces. Crystal faces (110), (101) and (001) are the length, width and thickness of MH, respectively. It can be seen from Table 2 that MH on the surface of CFAMH shows a flake shape, and the thickness of flake MH is 13.4 nm, confirming the SEM results.

### 3.4. Fourier Translation Infrared Spectroscopy (FTIR) Analysis

**Figure 5:**
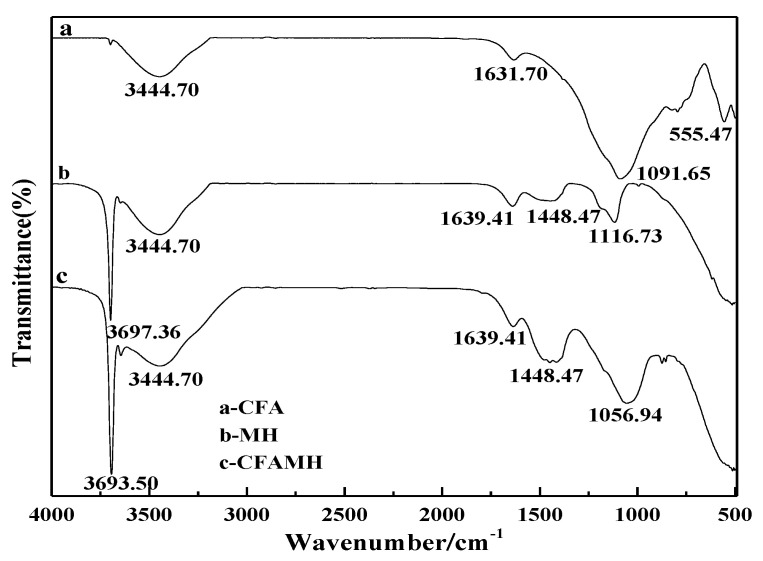
Fourier translation infrared spectroscopy (FTIR) spectra of CFA, MH and CFAMH.

Figure 5 is the FTIR spectra of CFA, MH and CFAMH. The bands at 3440.70, 555.47, 1631.7 and 1091.65 cm^−1^ were attributed to the O–H stretching vibration characteristic adsorption peak, Si–O bending vibration, O–H bending vibration and Si–O–Si asymmetric stretching, respectively [29]. The peaks of MH shown in Figure 5 matched well with the data reported for MH powders. The major band at 3697.36 cm^−1^ is due to the antisymmetric stretching vibration of the O–H band [18]. The adsorption band of this peak is strong, sharp and narrow, and it is a typical hydrogen and oxygen bond [18]. No hydrogen bond exists, indicating that there are many free O–H on the surface of Mg(OH)_2_, which is beneficial to the heterogeneous nucleation reaction. The peak at 1448.47 cm^−1^ is most likely due to O–H bending vibration bands of MH [18]. The bands at 600 and 435 cm^−1^ were ascribed to Mg–O bonds [18]. Comparing CFA and CFAMH, the band at 3693.50 cm^−1^ is due to the antisymmetric stretching vibration of the O–H band. The peak at 1448.47 cm^−1^ is due to O–H bending vibration bands of MH, and the intensity increased. The band at 555.47 cm^−1^ disappeared. The peak at 1631.70 cm^−1^ shifted to 1639.41 cm^−1^ and the intensity increased. Such a change shows that the free hydroxyl of CFA decreased while the associative hydroxyl increased, attributing to the Si-OH of the CFA reaction with O–H of Mg(OH)_2_ to form Si–O–Mg–OH.

### 3.5. The Preparation Mechanism of Core-Shell CFAMH

**Figure 6:**
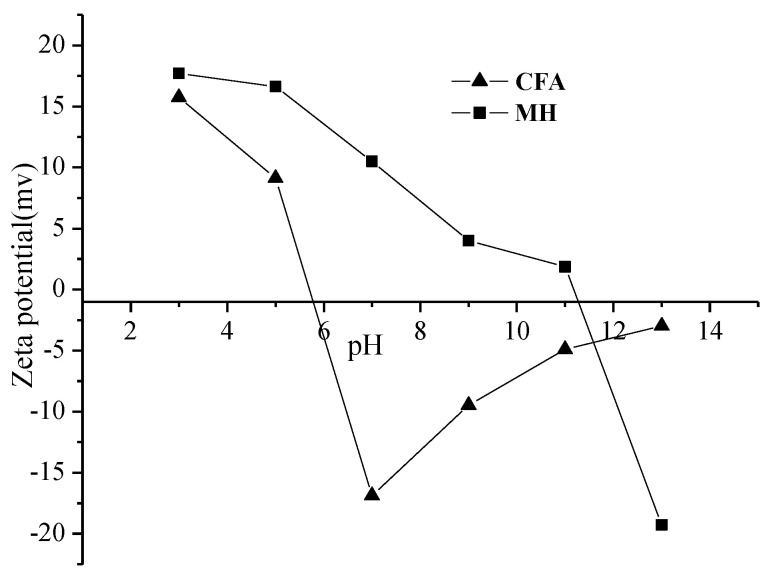
Zeta potential of CFA and MH under different pH values.

Zeta potential of CFA and MH under different pH values is shown in Figure 6. In the process of MgSO_4_-CFA-NaOH, magnesium sulfate will react with sodium hydroxide to precipitate magnesium hydroxide. When pH = 10, CFA has a negative surface charge, while MH particles have a positive charge. The two particles have the tendency to attract each other and neutralize the surface charge. Under the action of electrostatic gravity, the binding force between CFA and MH is enhanced, and the possibility of the same particles to form aggregates is also reduced. Although there is electrostatic attraction between CFA and MH, the electrostatic force is very small. As can be seen from Figure 5, both CFA and MH contain a large number of hydroxyl groups. The binding force of Si–O–Mg–OH chemical bond produced by hydroxyl condensation is much greater than that of electrostatic force, thus forming a stable core-shell structure.

Under alkaline conditions, the Si–O–Si and Si–O–Al bonds on the CFA surface break, resulting in multiple activation points, which are conducive to the free Mg^2+^ and OH^–^ participating in the reaction to generate precipitation and nucleation at the active site. When the actual radius of MH is smaller than the critical nucleus radius, the nucleus will be dissolved again. When the crystal nucleus radius is greater than the critical radius of the nucleus, MH will precipitate from solution, reducing the degree of supersaturation of the solution, so magnesium sulfate and sodium hydroxide reaction ion should be added constantly to stabilize the system from metastable state, thus reducing the suspension Gibbs-free energy. Crystal nucleus under the condition of water bath heating began to spontaneously absorb ions in the solution from a higher concentration area. The crystal nucleus grows in the interface along the steps of the tangential and normal continuous deposition and adsorption, and the old interfaces are covered while new interfaces are constantly produced.

**Figure 7:**
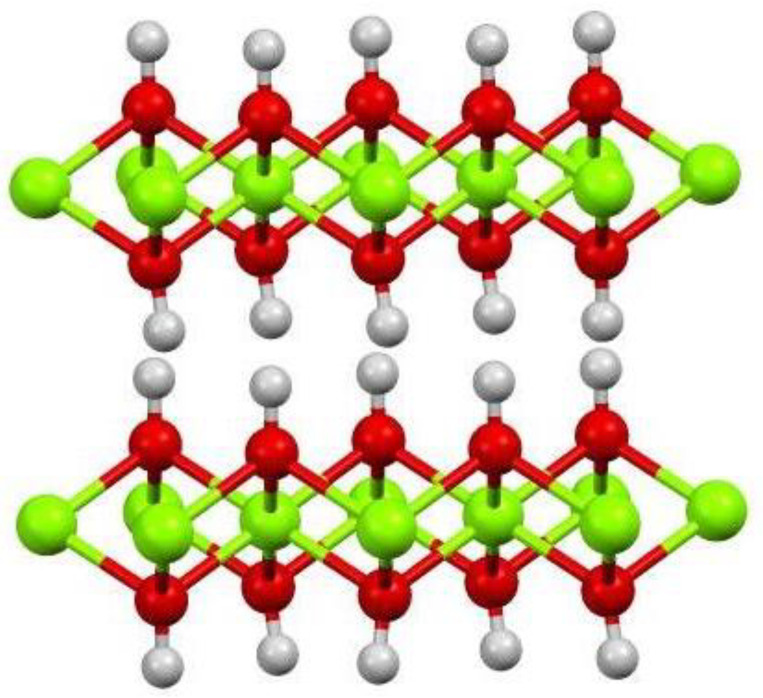
Three-dimensional (3D) structure of flake MH.

**Figure 8:**
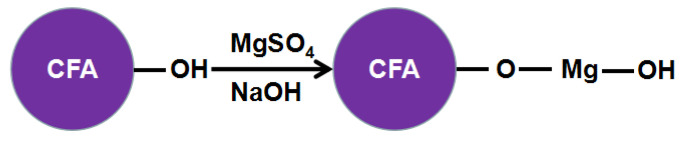
Preparation mechanism of CFAMH composites.

MH crystals have many shapes including spherical, rod-shaped, acicular, fibrous, flake, circular plate and elliptical flake form. From XRD and SEM results, the MH layer has irregular thin plate shape, and part of the vertex angle circle. The (101) surface has the sharpest peak and the most complete crystal shape. The 001 face has the slowest growth rate while the 110 face has the fastest growth, and the crystal grows along the (110) face, indicating that the MH growth pattern is two-dimensional extension type, which is relevant to the connection mode of the growth of primitive one. Although the connection mode of MH crystal structure is to share the apex angle, it is not connected in the way of an octahedron, but in the way of three Mg^2+^ connected by a hydroxyl group, and only grows along the X and Y axis. This connection has high stability, resulting in faster growth of the 110 face, which makes this face difficult to expose, while the vertical axis direction 001 face has no chemical bond connection and poor stability, resulting in low growth or no growth, which makes the 001 face easy to expose, and the thickness is small, resulting in the flake-shaped crystal with only 13.4 nm thickness (Figure 7). When magnesium sulfate and sodium hydroxide solutions were added to the CFA suspension at a steady rate at 90 °C, the crystals grow in the unforced system, the ions in the solution are in the supersaturated state, the crystal nucleus precipitated from the solution spontaneously, and the continuously added magnesium sulfate and sodium hydroxide solutions will continue to supplement the ions concentration in the solution, making the solution concentration meet the nucleation precipitation. Grain interface energy reduction and diffusion effect provide the driving force for the crystal nucleus to continue to grow, causing the crystal growth mode of each face to expose fully. In view of the above analysis, the synthesis mechanism of CFAMH composites can be illustrated by Figure 8.

### 3.6. Adsorption Tests

#### 3.6.1. Comparison of CFA, MH and CFAMH for Adsorption of Heavy Metal Ions

The adsorption results of CFA, MH and CFAMH for removal of heavy metals are shown in Table 3. *C*_0_ is the initial concentration of heavy metal ions, *C*_t_(mg/L) is the concentration of heavy metal ions at time t. As shown, CFA produces little removal efficiency for Cu^2+^, Zn^2+^ and Ni^2+^, while the CFAMH exhibits much higher removal efficiency at more than 80% for Cu^2+^, Zn^2+^ and Ni^2+^, which is due to the increase of specific surface area and more active groups of OH generated from analysis of FTIR. The removal efficiency of heavy metals on CFA, MH and CFAMH follows an order of Cu^2+^ > Zn^2+^ > Ni^2+^. When Cu^2+^, Zn^2+^ and Ni^2+^ are present simultaneously in the same solution, it also follows the same order of Cu^2+^ > Zn^2+^ > Ni^2+^. The reason for this is that the removal of Cu^2+^, Zn^2+^ and Ni^2+^ follows the mechanism of precipitation and substitution [14] and the solubility product constant (Ksp) follows the order of Mg(OH)_2_(8.19 × 10^−12^) > Ni(OH)_2_(1.16 × 10^−16^) > Zn(OH)_2_(1.2 × 10^−17^) > Cu(OH)_2_ (2.2 × 10^−20^). It is worthwhile to mention that MH also gives low removal efficiency in acidic solution. What caused it is that a little amount of MH powder was left after reacting with the acid. MH powder is difficult to be separated from the solution due to its nano size. In ICP-OES detection, nitrate acid was added to the solution to prevent precipitation, and the powder left in the liquid will react with the nitrate acid, leading to the increase of heavy metals’ concentration. Viewing the above analysis, in the following experiments, only the adsorption performance of heavy metal ions onto CFAMH was conducted.

#### 3.6.2. Adsorption Test of CFAMH for Cu^2+^, Zn^2+^ and Ni^2+^

Table 4, Table 5 and Table 6 are the adsorption results of CFAMH for Cu^2+^, Zn^2+^ and Ni^2+^. The national first grade emission standard for Cu^2+^, Zn^2+^ and Ni^2+^ concentration is 0.5, 5 and 1 mg/L respectively, and the pH value is required to be 6–9.

Table 4 shows that when the initial concentration of Cu^2+^ is 50 mg/L, the adsorbent concentration of CFAMH is 1 g/L, the initial pH value is 4, the adsorption time is 1440 min, the equilibrium concentration of Cu^2+^ is 0.42 mg/L and the equilibrium pH value is 6.42, which meets the first order of “National Overall Discharge Standard of Sewage (GB 8978-1996)”. When the adsorbent concentration of CFAMH is 2 g/L, the initial pH value is 4, the adsorption time is 120 min, the equilibrium concentration of Cu^2+^ is 0.44 mg/L and the equilibrium pH value is 7.68, which also meets the first order of GB 8978-1996. When the initial pH value is 2, the adsorbent concentration of CFAMH should be increased to 4 g/L to meet the first order of GB 8978-1996, that is because CFAMH firstly neutralize the H^+^ in the solution, and then adsorb Cu^2+^. When the initial concentration of Cu^2+^ is 35 mg/L, the adsorbent concentration of CFAMH is 1 g/L, the initial pH value is 4, the adsorption time is 1080 min, the equilibrium concentration of Cu^2+^ is 0.38 mg/L and the equilibrium pH value is 6.31. When the initial pH value is 2, the adsorbent concentration of CFAMH should be increased to 3 g/L to meet the first order of GB 8978-1996, and the adsorption time is reduced to 120 min. When the initial concentration of Cu^2+^ is 20 mg/L, the adsorbent concentration of CFAMH is 1 g/L, the initial pH value is 4, the adsorption time is 20 min, the equilibrium concentration of Cu^2+^ is 0.36 mg/L and the equilibrium pH value is 6.67. When the initial pH value is 2, the adsorbent concentration of CFAMH should be increased to 2 g/L to meet the first order of GB 8978-1996, while the adsorption time should remain constant.

Table 5 shows that when the initial concentration of Zn^2+^ is 50 mg/L, the adsorbent concentration of CFAMH is 6 g/L, the initial pH value is 2, the adsorption time is 240 min, the equilibrium concentration of Zn^2+^ is 4.3 mg/L and the equilibrium pH value is 7.84. When the initial concentration of Zn^2+^ is 35 mg/L, the adsorbent concentration of CFAMH is 3 g/L, the initial pH value is 2, the adsorption time is 60 min, the equilibrium concentration of Zn^2+^ is 3.2 mg/L and the equilibrium pH value is 7.62. When the initial concentration of Zn^2+^ is 20 mg/L, the adsorbent concentration of CFAMH is 2 g/L, the initial pH value is 2, the adsorption time is 20 min, the equilibrium concentration of Zn^2+^ is 2.4 mg/L and the equilibrium pH value is 7.13, which meets the second order of “National Overall Discharge Standard of Sewage (GB 8978-1996)”.

Table 6 shows that when the initial concentration of Ni^2+^ is 50 mg/L, the adsorbent concentration of CFAMH is 13 g/L, the initial pH value is 2, the adsorption time is 240 min, the equilibrium concentration of Ni^2+^ is 0.72 mg/L and the equilibrium pH value is 8.62. When the initial concentration of Ni^2+^ is 35 mg/L, the adsorbent concentration of CFAMH is 9.5 g/L, the initial pH value is 2, the adsorption time is 60 min, the equilibrium concentration of Ni^2+^ is 0.32 mg/L and the equilibrium pH value is 8.31. When the initial concentration of Ni^2+^ is 20 mg/L, the adsorbent concentration of CFAMH is 6 g/L, the initial pH value is 2, the adsorption time is 20 min, the equilibrium concentration of Ni^2+^ is 0.28 mg/L and the equilibrium pH value is 8.43, which meets the first order of “National Overall Discharge Standard of Sewage (GB 8978-1996)”.

In addition, Table 4, Table 5 and Table 6 show that the equilibrium concentration shows a decreasing trend with the decreasing initial concentration, which is similar to the research reported before [30]. This can be explained by the increase in the number of heavy metals compared with the number of active sites on the CFAMH adsorbent.

## 4. Conclusions

SEM, XRD, FTIR and BET results showed that CFAMH nanocomposite with 13.4 nm flake MH was successfully prepared with a heterogeneous nucleation method. Si–O–Mg–OH bonds formed. Electrostatic attraction and hydroxyl condensation led to the formation of CFAMH. CFAMH nanocomposite was used for adsorption of Cu^2+^, Zn^2+^ and Ni^2+^ from acidic solution. The removal efficiency of the heavy metal ions on CFA, MH and CFAMH follows an order of Cu^2+^ > Zn^2+^ > Ni^2+^. K_sp_ is an important constant for the removal order of heavy metals on FA, CFAMH and MH. CFAMH nanocomposite can be a cheap material for removing heavy metal ions from acidic wastewater.

## Figures and Tables

**Table 1:** Specific surface areas and pore characteristics of fly ash (FA), calcined fly ash (CFA) and calcined fly ash coated with magnesium hydroxide (CFAMH) powders.

Samples	S_BET_ (m^2^/g)	V_total_ (cm^3^/g)	V_meso_ (cm^3^/g)	V_mac_ (cm^3^/g)
FA	3.7	0.0115	0.0113	0.0002
CFA	2.5	0.0093	0.0091	0.0002
CFAMH	31.0	0.0329	0.0325	0.0004

**Table 2:** Calculating data of average crystallite sizes of MH on CFAMH from the Scherrer equation.

Crystal Face	2θ (°)	h (mm)	β (rad)	D (nm)
(101)	37.88	601.13	0.00672	20.99
(001)	18.32	314.34	0.01012	13.4
(110)	58.54	278.00	0.00375	40.83

**Table 3:** The adsorption results of calcined fly ash (CFA), calcined fly ash coated with magnesium hydroxide (CFAMH) and magnesium hydroxide (MH) for removal of heavy metals.

Samples (Adsorbent Dosage) (g/L)	Heavy Metals	*C_0_* (mg/L)	InitialpH	Time (min)	Equilibrium pH	Filtration Time (min)	*C_t_* (mg/L)	Removal Efficiency (%)
CFA (2 g/L)	Cu^2+^	35	2	120	3.20	10	34.61	1.11
	Zn^2+^	35	2	120	3.13	10	34.78	0.63
	Ni^2+^	35	2	120	3.15	10	34.86	0.4
CFAMH (2 g/L)	Cu^2+^	35	2	120	6.66	10	3.03	91.34
	Zn^2+^	35	2	120	6.8	10	3.7	89.43
	Ni^2+^	35	2	120	7.01	10	4.1	88.29
	Cu^2+^/ Zn^2+^/ Ni^2+^	11.67/11.67/ 11.67	2	120	7.08	10	0.92/ 8.21/ 11.40	92.12/ 29.65/ 2.31
MH (0.6 g/L)	Cu^2+^	35	2	120	4.51	10	28.3	19.14
	Zn^2+^	35	2	120	4.87	10	29.7	15.14
	Ni^2+^	35	2	120	4.91	10	31.4	10.29
	Cu^2+^/ Zn^2+^/ Ni^2+^	11.67/11.67/ 11.67	2	120	4.96	10	8.64/ 10.61/ 11.24	25.96/ 8.6/ 3.68

**Table 4:** The adsorption results of CFAMH for Cu^2+^.

Initial Concentration of Cu^2+^ (mg/L)	Adsorbent Concentration (g/L)	Initial pH Value	Time (min)	Equilibrium Concentration (mg/L)	Equilibrium pH Value
50	1	4	15	30.14	/
50	1	4	120	20.88	/
50	1	4	180	15.86	/
50	1	4	600	5.24	/
50	1	4	1080	0.87	/
50	1	4	1440	0.42	6.42
50	2	4	120	0.44	7.68
50	4	2	240	0.36	8.56
35	1	4	15	13.33	/
35	1	4	60	8.07	/
35	1	4	120	4.25	/
35	1	4	180	2.34	/
35	1	4	1080	0.38	6.31
35	3	2	120	0.11	7.94
20	1	4	20	0.36	6.67
20	2	2	20	0.42	6.56

**Table 5:** The adsorption results of CFAMH for Zn^2+^.

Initial Concentration of Zn^2+^(mg/L)	Adsorbent Concentration (g/L)	Initial pH Value	Time (min)	Equilibrium Concentration (mg/L)	Equilibrium pH Value
50	6	2	240	4.3	7.84
35	3	2	60	3.2	7.62
20	2	2	20	2.4	7.13

**Table 6:** The adsorption results of CFAMH for Ni^2+^.

Initial Concentration of Ni^2+^ (mg/L)	Adsorbent Concentration (g/L)	Initial pH Value	Time (min)	Equilibrium Concentration (mg/L)	Equilibrium pH Value
50	13	2	240	0.72	8.62
35	9.5	2	60	0.32	8.31
20	6	2	20	0.28	8.43
